# Core-Symptom-Defined Cortical Gyrification Differences in Autism Spectrum Disorder

**DOI:** 10.3389/fpsyt.2021.619367

**Published:** 2021-04-20

**Authors:** Mingmin Ning, Cuicui Li, Lei Gao, Jingyi Fan

**Affiliations:** ^1^Department of Pediatrics, Zhongnan Hospital of Wuhan University, Wuhan, China; ^2^Department of Radiology, Zhongnan Hospital of Wuhan University, Wuhan, China

**Keywords:** cortical folding, autism, brain development, language, meta-analysis

## Abstract

Autism spectrum disorder (ASD) is a heterogeneous disease that is characterized by abnormalities in social communication and interaction as well as repetitive behaviors and restricted interests. Structural brain imaging has identified significant cortical folding alterations in ASD; however, relatively less known is whether the core symptoms are related to neuroanatomical differences. In this study, we aimed to explore core-symptom-anchored gyrification alterations and their developmental trajectories in ASD. We measured the cortical vertex-wise gyrification index (GI) in 321 patients with ASD (aged 7–39 years) and 350 typically developing (TD) subjects (aged 6–33 years) across 8 sites from the Autism Brain Imaging Data Exchange I (ABIDE I) repository and a longitudinal sample (14 ASD and 7 TD, aged 9–14 years in baseline and 12–18 years in follow-up) from ABIDE II. Compared with TD, the general ASD patients exhibited a mixed pattern of both hypo- and hyper- and different developmental trajectories of gyrification. By parsing the ASD patients into three subgroups based on the subscores of the Autism Diagnostic Interview—Revised (ADI-R) scale, we identified core-symptom-specific alterations in the reciprocal social interaction (RSI), communication abnormalities (CA), and restricted, repetitive, and stereotyped patterns of behavior (RRSB) subgroups. We also showed atypical gyrification patterns and developmental trajectories in the subgroups. Furthermore, we conducted a meta-analysis to locate the core-symptom-anchored brain regions (circuits). In summary, the current study shows that ASD is associated with abnormal cortical folding patterns. Core-symptom-based classification can find more subtle changes in gyrification. These results suggest that cortical folding pattern encodes changes in symptom dimensions, which promotes the understanding of neuroanatomical basis, and clinical utility in ASD.

## Introduction

Autism spectrum disorder (ASD) is a neurodevelopmental, heterogeneous disease that is characterized by abnormalities in social communication and interaction as well as repetitive behaviors and restricted interests (1). Structural brain imaging has revealed prominent morphological and developmental alterations in ASD [for a review, please see (2)]. However, relatively less is known whether the core symptoms are related to neuroanatomical differences.

One of the most prominent morphological changes in ASD is cortical gyrification. Gyrification refers to the process of cortical convolution (3) and has been actively investigated in ASD due to its role encoding early neurodevelopment (3, 4). Previous studies have reported altered gyrification in different ASD populations (5–7). For example, an early study reported higher gyrification in ASD individuals during childhood and adolescence (8). More recent studies identified increased gyrification in a number of cortical foci including paracentral (6, 9, 10), cingulate (10, 11), occipital, and temporal cortices (11, 12) in children and adolescents with ASD; other studies also found decreased gyrification in the parietal cortex (7, 13) in adults with ASD and temporo-parietal junction in children with ASD (14, 15). Despite the existing findings, there is a lack of consistent gyrification alterations across the ASD studies.

Most previous studies, however, treated ASD as a singular disease entity, ignoring the symptom heterogeneity (symptom weights or symptom-defined subgroups). We know that the heterogeneity, i.e., impaired in reciprocal social interaction (RSI), communication abnormalities (CA), and restricted, repetitive, and stereotyped patterns of behavior (RRSB), also represents cognition and behavior dimensions. Each dimension reflects brain function in social interaction, communication (language), precise motor planning, and unusual interests. Although overlaps may exist, these brain functions are subserved by different regions (circuits). For example, much of previous neuroimaging work identified dimensional involvements as neural markers for ASD, including superior temporal (2) and inferior frontal (16) regions for language processing, fusiform face area regions (17) for social communication, temporal and fusiform regions for social interactions (12), and (pre)motor regions for restricted repetitive behaviors (18). Thus, a symptom-oriented stratification may yield a more sensitive characterization of morphological alterations.

In this study, we sought to: (1) investigate symptom-defined subgroup gyrification alterations in ASD, (2) map regionally developmental trajectories of gyrification in ASD, and (3) locate the brain co-activation meta-analytical maps corresponding to the symptom dimensions using the NeuroSynth database (19). We hypothesized that: (1) the ASD individuals showed symptom-specific differences on cortical gyrification compared with their matched typically developing (TD) controls, (2) the general ASD and the three subgroups would demonstrate regional-specific developmental trajectories of gyrification, and (3) the atypical patterns of gyrification in the three subgroups would be consistent with the meta-analysis identified co-activation maps.

## Materials and Methods

### Subjects

All magnetic resonance imaging (MRI) and phenotypic data are available from the Autism Brain Imaging Data Exchange (ABIDE) repository (http://fcon_1000.projects.nitrc.org/indi/abide/), which is hosted by the 1000 Functional Connectomes Project/International Neuroimaging Data-sharing Initiative (INDI). The ABIDE I consists of 1,112 subjects, including 539 ASD and 573 TD individuals (ages 7–64 years) from 17 international institutions (see http://fcon_1000.projects.nitrc.org for more information). Patients had been verified the Diagnostic and Statistical Manual of Mental Disorders, fourth edition, text revision (DSM-IV-TR) diagnosis of ASD, established by combining expert clinical opinion and Autism Diagnostic Interview—Revised (ADI-R)/the Autism Diagnostic Observation Schedule. We first screened ABIDE I and selected only eligible sites of data. Inclusion criteria were sites with: (1) successfully pre-processed T1-weighted anatomical images, (2) age- and gender-matched between ASD and controls, (3) a minimum of 20 subjects (10 ASD/10 TD), and (4) subjects with complete ADI-R scores. A total of 671 participants (321 ASD/350 TD) from 8 research centers were included. Furthermore, we employed the longitudinal data from the University of California Los Angeles (UCLA) in ABIDE II to discover the developmental trajectory of gyrification in ASD. Demographic and clinical information are summarized in Tables 1-1, 1-3.

Table 1.1Participant demographics.

**Table:** 

**Variables**			**NYU**	**UCLA**	**UM**	**KKI**	**PITT**	**STANFORD**	**TRINITY**	**YALE**	**Total**
ASD and TD	Subjects (*N*)	ASD (TD)	79 (105)	53 (45)	65 (69)	22 (33)	30 (27)	20 (20)	24 (23)	28 (28)	321 (350)
	Age (years)	ASD	14.52 ± 6.97	13.04 ± 2.46	13.26 ± 2.41	10.01 ± 1.45	18.93 ± 7.20	9.96 ± 1.59	17.28 ± 3.57	17.28 ± 3.57	13.58 ± 4.51
		TD	15.81 ± 6.25	12.69 ± 1.92	14.09 ± 3.54	10.16 ± 1.26	18.88 ± 6.64	9.95 ± 1.60	17.17 ± 3.79	17.17 ± 3.79	14.12 ± 4.85
	Gender M (F)	ASD	68 (11)	47 (6)	56 (9)	18 (4)	26 (4)	16 (4)	24 (0)	20 (8)	275 (46)
		TD	79 (26)	39 (6)	54 (15)	24 (9)	23 (4)	16 (4)	23 (0)	20 (8)	288 (72)
	ADI-R-Score	Social	19.19 ± 5.61	20.19 ± 5.16	19.44 ± 4.89	20.33 ± 6.00	20.54 ± 3.76	20.40 ± 5.40	20.29 ± 6.06	21.95 ± 5.52	19.99 ± 5.28
		Verbal	15.87 ± 4.40	16.33 ± 4.63	15.56 ± 3.67	15.48 ± 5.13	15.65 ± 3.95	15.90 ± 5.12	16.00 ± 4.98	18.05 ± 4.08	16.01 ± 4.39
		RRB	5.69 ± 2.65	7.25 ± 2.48	6.33 ± 2.51	5.86 ± 1.82	6.31 ± 2.22	5.84 ± 2.52	5.42 ± 2.69	5.27 ± 2.62	6.11 ± 2.55
	Statistics	Age	*t* = −1.32, *p* = 0.189	*t* = 0.18, *p* = 0.86	*t* = −1.86, *p* = 0.07	*t* = −0.41, *p* = 0.69	*t* = −0.03, *p* = 0.98	*t* = −0.02, *p* = 0.99	*t* = 0.10, *p* = 0.92	*t* = 0.09, *p* = 0.93	*t* = −1.46, *p* = 0.15
		Gender	*X*^2^ = 3.30, *p* = 0.07	*X*^2^ = 0.09, *p* = 0.76	*X*^2^ = 1.42, *p* = 0.23	*X*^2^ = 0.60, *p* = 0.44	*X*^2^ = 0.03, *p* = 0.87	–	–	–	*t* = 3.22, *p* = 0.07
RSI and TD	Subjects (N)	RSI (TD)	34 (67)	25 (45)	33 (59)	14 (25)	13 (16)	14 (20)	13 (18)	13 (24)	159 (274)
	Age (years)	RSI	13.05 ± 4.73	13.28 ± 2.46	13.03 ± 2.21	10.26 ± 1.46	18.32 ± 7.14	9.89 ± 1.63	16.09 ± 2.82	12.45 ± 2.97	13.20 ± 4.09
		TD	13.87 ± 4.30	12.96 ± 1.92	13.75 ± 2.62	10.37 ± 1.31	18.69 ± 7.38	9.95 ± 1.60	15.95 ± 2.87	12.52 ± 2.57	13.39 ± 3.86
	Gender M (F)	RSI	31 (3)	23 (2)	25 (8)	13 (1)	10 (3)	11 (3)	13 (0)	10 (3)	136 (23)
		TD	63 (4)	39 (6)	45 (14)	24 (1)	13 (3)	16 (4)	18 (0)	17 (7)	235 (39)
	ADI-R-Score	Social	21.76 ± 4.59	21.80 ± 4.48	22.18 ± 3.50	22.21 ± 5.32	21.15 ± 3.39	20.64 ± 5.42	22.62 ± 5.44	23.46 ± 4.20	20.82 ± 5.16
		Verbal	15.48 ± 4.00	25.88 ± 3.83	15.48 ± 3.44	15.21 ± 4.41	15.85 ± 3.87	15.21 ± 5.38	15.08 ± 4.82	17.31 ± 3.20	15.72 ± 4.22
		RRB	4.70 ± 2.23	6.32 ± 2.54	5.70 ± 1.86	6.00 ± 1.96	5.38 ± 1.80	5.00 ± 1.96	4.85 ± 1.57	5.92 ± 2.90	5.64 ± 2.33
	Statistics	Age	*t* = −0.87, *p* = 0.38	*t* = 0.60, *p* = 0.55	*t* = −1.33, *p* = 0.19	*t* = −0.25, *p* = 0.80	*t* = −0.14, *p* = 0.89	*t* = −0.12, *p* = 0.90	*t* = 0.14, *p* = 0.89	*t* = −0.07, *p* = 0.94	*t* = −0.49, *p* = 0.62
		Gender	*X*^2^ = 0.29, *p* = 0.59	*X*^2^ = 0.45, *p* = 0.50	*X*^2^ = 0.02, *p* = 0.90	*X*^2^ = 0.18, *p* = 0.67	*X*^2^ = 0.08, *p* = 0.78	*X*^2^ = 0.01, *p* = 0.92	–	*X*^2^ = 0.16, *p* = 0.69	*X*^2^ = 0.013, *p* = 0.91

**Table:** 

	**Variables**		**NYU**	**UCLA**	**UM**	**Total**
RRSB and TD	Subjects (*N*)	RRSB (TD)	13 (38)	16 (32)	16 (47)	45 (117)
	Age (years)	RRSB	14.79 ± 7.39	12.80 ± 2.55	12.85 ± 2.63	13.45 ± 4.67
		TD	13.84 ± 5.46	13.05 ± 1.78	13.54 ± 2.74	13.50 ± 3.67
	Gender M (F)	RRSB	12 (1)	15 (1)	15 (1)	42 (3)
		TD	37 (1)	28 (4)	46 (1)	111 (6)
	ADI-R-Score	Social	15.00 ± 4.93	17.13 ± 5.23	17.56 ± 5.05	16.67 ± 5.08
		Verbal	12.69 ± 3.71	14.06 ± 4.67	14.13 ± 2.94	13.69 ± 3.81
		RRB	8.38 ± 2.57	8.88 ± 1.86	8.69 ± 2.15	8.67 ± 2.14
	Statistics	Age	*t* = 0.49, *p* = 0.62	*t* = −0.39, *p* = 0.70	*t* = −0.89, *p* = 0.38	*t* = −0.08, *p* = 0.94
		Gender	*X*^2^ = 0.66, *p* = 0.42	*X*^2^ = 0.45, *p* = 0.50	*X*^2^ = 0.66, *p* = 0.42	*X*^2^ = 0.12, *p* = 0.73
CA and TD	Subjects (N)	CA (TD)	22 (66)	10 (35)	17 (44)	49 (145)
	Age (years)	CA	13.39 ± 5.49	13.00 ± 2.75	13.42 ± 2.73	13.32 ± 4.14
		TD	13.96 ± 5.11	13.00 ± 2.10	14.66 ± 2.67	13.94 ± 3.92
	Gender M (F)	CA	17 (5)	8 (2)	16 (1)	41 (8)
		TD	48 (18)	31 (4)	43 (1)	122 (23)
	ADI-R-Score	Social	17.73 ± 5.62	20.60 ± 5.02	15.41 ± 4.39	17.51 ± 5.34
		Verbal	18.41 ± 4.27	20.80 ± 3.65	17.06 ± 4.34	18.43 ± 4.31
		RRB	5.59 ± 2.34	6.70 ± 1.89	5.24 ± 2.46	5.69 ± 2.32
	Statistics	Age	*t* = −0.44, *p* = 0.66	*t* = 0.00, *p* = 1	*t* = −1.60, *p* = 0.12	*t* = −0.94, *p* = 0.35
		Gender	*X*^2^ = 0.18, *p* = 0.67	*X*^2^ = 0.50, *p* = 0.48	*X*^2^ = 0.50, *p* = 0.48	*X*^2^ = 0.94, *p* = 0.006

*ASD, patients with autism spectrum disorder; TD, typically developing controls; RSI, impairments in reciprocal social interaction as the most serious in the three symptoms; CA, communication abnormalities as the most serious in the three symptoms; RRSB, stereotyped patterns of behavior as the most serious in the three symptoms*.

### MRI Data

A total of 1,154 high-resolution T1-weighted images were downloaded from 8 sites in the ABIDE website (http://fcon_1000.projects.nitrc.org/indi/abide/). The MRI scanner and parameter details can be seen in the website (http://fcon_1000.projects.nitrc.org/indi/abide/) or Supplementary Table 1.

### Data Processing

Data processing was completed using the Statistical Parametric Mapping 12 (http://www.fil.ion.ucl.ac.uk/spm/software/spm12/) and Computational Anatomy Toolbox 12 (CAT12.6) toolbox (http://dbm.neuro.uni-jena.de/cat/). The CAT12 toolbox provides a pipeline for surface-based morphometry, allowing for the extraction of the cortical surface with a novel algorithm and multiple morphometric parameters including cortical thickness and gyrification index (GI) (14, 20, 21). Thus, we employed the default parameters of CAT12 for this pre-processing procedure, except for tissue probability map (TPM), to process MRI data, and the specific steps are as follows. We visually screened T1-weighted images for tissue segmentation. Local maxima were then projected to gray matter voxels by using a successor relationship described by the white matter (WM) distance that equals cortical thickness with age-specific brain TPMs (7–12, 1–18, and 18–65 years old). This projection-based method (22) also included partial volume correction, sulcal blurring, and sulcal asymmetries without sulcus reconstruction. The correction of the projection-based thickness was completed by spherical harmonics. For spherical registration of inter-participant, an adapted diffeomorphic anatomical registration through the exponentiated lie algebra (DARTEL) algorithm was applied to the surface based on spherical mapping of the cortical surface (23).

We computed the vertex-wise GI based on the absolute mean curvature approach (23). The mean curvature values were computed from each vertex within 3 mm from a certain point as the local absolute mean curvature of this central surface (the surface between the gray matter/cerebrospinal fluid border and the gray matter/WM boundary). The GI surfaces were then spatially smoothed with 12 mm full-width at half maximum (FWHM), which resulted in smoothed GI maps for further statistical analyses.

Prior to statistical analysis, all GI maps were visually inspected, and individuals with obvious segmentation or reconstruction defects and those who were outside the standard deviation of the sample by 2 times were also excluded.

### Core-Symptom-Based Subgroups of ASD

**Figure 1:**
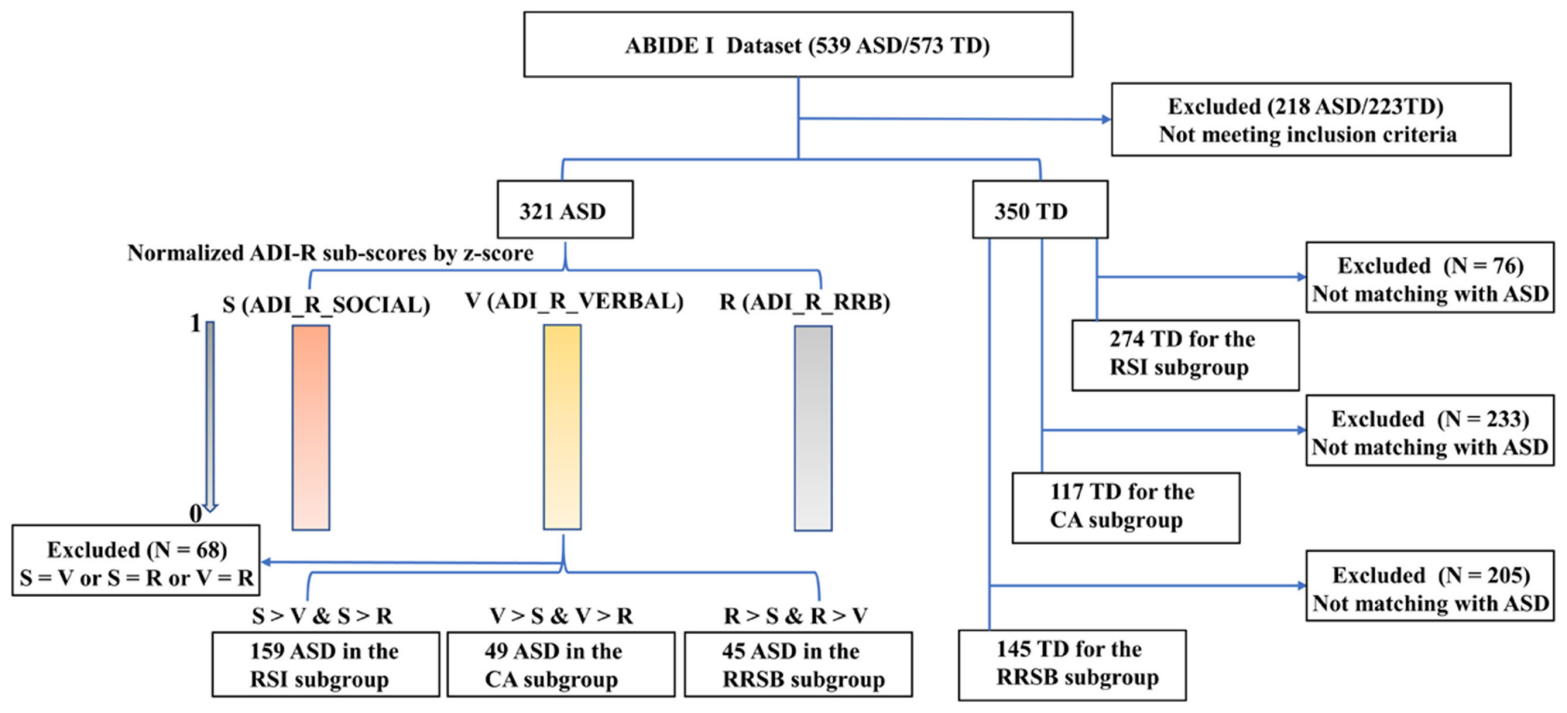
Main steps for subgroups divided for ASD. ADI-R-SOCIAL: ADI-R-SOCIAL-TOTAL-A, Reciprocal Social Interaction Subscore (A) for Autism Diagnostic Interview—Revised; ADI-R-VERBAL: ADI-R-VREBAL-TOTAL-BV, Abnormalities in Communication Subscore (B) for Autism Diagnostic Interview—Revised; ADI-R-RRB: ADI-R-RRB-TOTAL-C, Restricted, Repetitive, and Stereotyped Patterns of Behavior Subscore (A) for Autism Diagnostic Interview—Revised. The ADI-R subscale scores were normalized for each subject using z-scores to obtain S, V, and R, respectively. Then, we divided the ASD subjects into three subgroups according to their normalized value weight, and each subject from the subgroups was matched to controls on gender, age, and site.

To explore whether patterns of cortical gyrification differed among ASD with different core symptoms, we divided the ASD cohorts into three subgroups: repaired RSI, CA, and RRSB dominant subgroups according to the ADI-R subscale scores. The ADI-R includes ADI-R-SOCIAL-TOTAL-A, ADI-R-VREBAL-TOTAL-BV, and ADI-R-RRB-TOTAL-C subscales, corresponding to the social, communication, and repetitive and stereotyped patterns of behavior domains. We normalized each subscale score of ASD subjects by z-scores and obtained S, V, and R (24). Then, we divided the ASD subjects into three subgroups according to their normalized value weights: (1) the RSI subgroup was dominated by the *S*-values (159 subjects included), (2) the CA subgroup was dominated by the *V*-values (49 included), and (3) the RRSB subgroup was dominated by the *R*-values (45 included). Finally, each subject from the subgroups was matched to controls on gender, age, and site (274 for the RSI group, 145 for the CA group, and 117 for the RRSB group). This practice is similar to Chen et al. (25). To maximize the sample size, some controls were reused in multiple subgroups. To examine the difference of the three subgroups, we conducted non-parametric tests for age and the subscores of ADI-R, one-way ANOVA-tests for Full IQ (FIQ), and Chi-square-test for gender with *post-hoc* Bonferroni correction in SPSS 20.0 (IBM SPSS Inc., Chicago, IL, USA). Detailed process and results can be found in Figure 1 and Tables 1-1, 1-2.

**Table 1.2:** Difference of participant demographics among three subgroups.

**Variables**	**Mean difference**	***p*-value**	**Std. mean difference**	***p*-value**	**Std. mean difference**	*p*-value^*^
	RSI vs. CA		RSI vs. RRSB		CA vs. RRSB	
ADI-R-SOCIAL	3.6170	0.0008	4.2457	<0.0001	0.6099	1.0000
ADI-R-VERBAL	−3.7679	<0.0001	2.5115	0.03607	5.0357	<0.0001
ADI-R-RRB	−0.2318	1.0000	−6.5652	<0.0001	−5.1856	<0.0001
Gender	–	1.0000	–	0.5861	–	0.4367
Age	−0.1271	1.0000	−0.1981	1.0000	−0.07097	1.0000
FIQ	−4.5811	0.2654	0.8296	1.0000	5.4107	0.3328

*ADI-R-SOCIAL, ADI-R-SOCIAL-TOTAL-A, Reciprocal Social Interaction Subscore (A) for Autism Diagnostic Interview—Revised; ADI-R-VERBAL: ADI-R-VREBAL-TOTAL-BV, Abnormalities in Communication Subscore (B) for Autism Diagnostic Interview—Revised; ADI-R-RRB: ADI-R-RRB-TOTAL-C, Restricted, Repetitive, and Stereotyped Patterns of Behavior Subscore (A) for Autism Diagnostic Interview—Revised; FIQ, Full IQ Standard Score*.

* *Bonferroni corrected p-values*.

### Brain Co-Activation Meta-Analytical Maps

Finally, we performed a meta-analysis to further determine the extent to which differences between the TD and the three subgroups of ASD reflect the symptom-related cortical projection dimensions. To examine the core symptoms of ASD-anchored brain regions (circuits), we searched for research terms in the NeuroSynth database (www.neurosynth.org) (19). The NeuroSynth database is a large meta-analytical synthesis database, utilizing information from text and Montréal Neurological Institute coordinates extracted in previous publications and posterior probability meta-analytical co-activation maps. Therefore, we searched three research terms including “social interaction(s),” “language,” and “repetitive and stereotyped behavior” and extracted each corresponding co-activation map.

### Statistical Analysis

Demographic and clinical variables were tested for between-group differences of gender using the Chi-square-test, independent two-sample *t*-tests for age, and other variables with SPSS 20.0.

For the GI, independent two-sample *t*-tests were performed between the ASD/RRSB/RSI/CA and TD groups, with age and gender as covariates in CAT12. To test the trajectories of gyrification in subgroups, the general ASD subgroups and the TD group, multiple quadratic regression analyses were calculated with gender as covariates CAT12. In the abovementioned analyses, we alleviated the site effects based on the linear mixed models (LMM) as described in Yan et al. (26). Meanwhile, we employed a longitudinal dataset to reveal the gyrification developmental trajectories of ASD and TD with paired *t*-tests in CAT12. Furthermore, to ensure that our results of vertex-wise comparisons are robust, we conducted region of interest (ROI)-wise analysis within the Desikan–Killiany 40 atlas (27) parcellation with LMM with site as a random factor in SPSS 20.0 (28). We tested the main effects of group, age (linear and quadratic), and scores of ADI-R and interaction effects of group and age on the GI in ROI-wise.

The Threshold-Free Cluster Enhancement (TFCE), 10,000 random non-parametric permutations, was used for multiple corrections, with vertex-wise *p* < 0.001 and cluster level *p* < 0.05, family-wise error (FWE) correction. Cohen's *d* was applied to indicate the effect size. The Desikan–Killiany (27) atlas was used for the significant cluster report.

## Results

### Demographics

Across the 1,112 subjects (539 ASD and 573 TD) in the ABIDE I, we excluded 441 subjects (218 ASD/223 TD) based on our inclusion criteria, and a total of 671 participants (321 ASD/350 TD, 8 sites) were included for symptom-based analyses (Table 1-1). The subjects were divided into three subgroups, i.e., RSI (159 ASD/274 TD), RRSB (45 ASD/117 TD), and CA (49 ASD/145 TD) (Table 1-1). In addition, the longitudinal data included 21 subjects from ABIDE II (14 ASD/7 TD) (Table 1-3). No significant difference in age and gender was found between each subgroup pair (*p* > 0.05). However, we found that the three subgroups are significantly different in the three symptom domains (*p* < 0.05) and no significant difference in age, gender, and FIQ (*p* > 0.05; Table 1-2).

**Table 1.3:** Participant demographics of UCLA.

**Variables**		**ASD**	**TD**	**Statistics**
Subjects (*N*)		14	7	–
Gender M (*F*)		13 (1)	7 (0)	*X*^2^ = 0.53, *p* = 0.47
Age (years)	Baseline	11.73 ± 1.88	12.23 ± 1.09	*t* = −0.93, *p* = 0.36
	Follow-up	14.57.73 ± 1.33	15.23 ± 1.14	*t* = −1.12, *p* = 0.27

### Between-Group Differences on Cortical Gyrification

**Figure 2:**
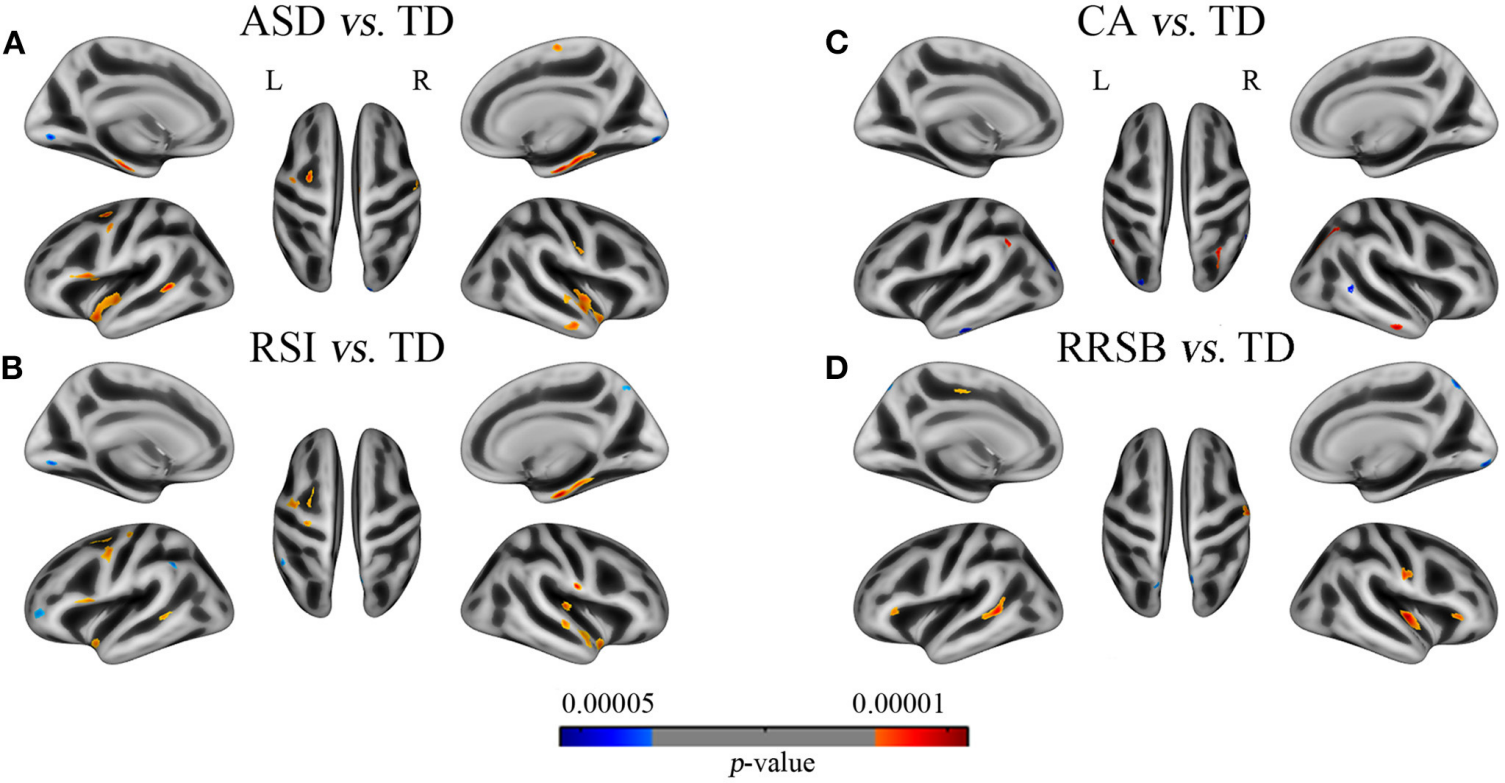
Differences of gyrification between patients with the general ASD, subgroups of ASD and corresponding TD groups. **(A)** ASD vs. TD: 11 clusters with increased GI in the bilateral superior temporal, parietal, parahippocampal, the left inferior frontal, and the right middle temporal cortices and 3 clusters with increased GI in the bilateral lingual and the right lateral occipital cortices; **(B)** RSI vs. TD: 12 clusters with increased GI in the bilateral parietal and superior temporal and the left frontal cortices and 4 clusters with decreased GI in the left supramarginal, middle frontal and lingual, and the right pre-cuneus cortices; **(C)** CA vs. TD: 3 clusters with increased GI in the bilateral inferior parietal and the right middle temporal cortices and 3 clusters with decreased GI in the left occipital and inferior temporal and the right middle temporal cortices; **(D)** RRSB vs. TD: 5 clusters with increased GI in the bilateral pars triangularis, the left superior temporal, and the right insula and post-central cortices and 1 cluster with decreased GI in the left superior parietal. Red color indicates ASD > TD, blue color indicates ASD < TD. The results were corrected by vertex level *p* < 0.001 and cluster level FWE *p* < 0.05.

To generate an overview of intergroup differences on the GI, we first compared the cortical GI between all ASD and TD (i.e., 321 ASD vs. 350 TD). As shown in Figure 2 and Table 2-1, the general ASD population exhibited both hyper- and hypo-gyrification across several cortical sites. More specifically, ASD demonstrated higher GI in the left pars opercularis, pre-central, superior temporal, parahippocampal, and caudal middle frontal cortices (*p* < 0.001) and the right temporal, pre-central, paracentral, post-central, and parahippocampal cortices (*p* < 0.001), as well as lower GI in the left lingual cortex and the right lateral occipital, lingual, and cuneus cortices (*p* < 0.001). These results were generally consistent with previous findings.

**Table 2.1:** General comparison of gyrification between ASD and TD.

***p*-value**	**Size**	**Overlap of atlas region**	***p*-value**	**Size**	**Overlap of atlas region**
**Left**			**Right**		
**ASD** **>** **TD**			**ASD** **>** **TD**		
0.00011	276	61% superior temporal	0.00007	294	72% superior temporal
		38% insula			28% insula
0.00013	134	96% pars opercularis	<0.00001	208	73% parahippocampal
		4% pre-central			26% entorhinal
0.00003	73	71% parahippocampal	0.00021	54	100% middle temporal
		29% entorhinal	0.00114	39	69% post-central
0.00001	53	100% bankssts			31% pre-central
0.00007	38	55% pre-central	0.00256	36	100% superior temporal
		34% caudal middle frontal	0.00031	30	100% paracentral
		11% superior frontal			
0.00061	28	100% pre-central			
**Left**			**Right**		
**ASD** **<** **TD**			**ASD** **<** **TD**		
0.00006	33	100% lingual	0.00005	30	87% lingual
					13% lateral occipital
			0.00006	27	59% lateral occipital
					41% cuneus

*Bankssts, the banks superior temporal sulcus. The results were corrected by vertex level p < 0.001 and cluster level FWE p < 0.05. The corrected and survived clusters were reported as % overlap with the Desikan–Killiany DK40 atlas*.

### Core-Symptom-Defined Between-Group Differences on Cortical Gyrification

To understand the core-symptom-based differences of the GI, we divided the general ASD into three subgroups based on the core symptoms and compared them with the corresponding TD groups. As expected, the three inter-subgroup comparisons yielded group-specific patterns of gyrification, though overlaps exist.

On the one hand, we found that the three subgroups showed abnormal GI in several brain regions that were absent in the general ASD group, compared with TD. The RSI subgroup exhibited significantly higher GI in the right transverse temporal cortex and lower GI in the left supramarginal and rostral middle frontal cortices (*p* < 0.001); the CA subgroup exhibited higher GI in the left supramarginal and inferior parietal cortices and the right inferior parietal cortex (*p* < 0.001) and lower GI in the left lateral occipital and inferior temporal cortices and the right middle temporal cortex (*p* < 0.001); the RRSB subgroup exhibited higher GI in the left pars triangularis and paracentral cortices (*p* < 0.001) and the right pars triangularis cortex and lower GI in the left superior parietal cortex (*p* < 0.001; Figure 2 and Table 2-2). On the other hand, there were several clusters that exhibited abnormal GI just in the general ASD group, including increased GI in the left parahippocampal and entorhinal cortices and decreased GI in the right paracentral cortex (*p* < 0.001).

**Table 2.2:** Comparison of gyrification between subgroups and TD.

***p*-value**	**Size**	**Overlap of atlas region**	***p*-value**	**Size**	**Overlap of atlas region**
**RSI vs. TD** ** Left hem: ASD** **>** **TD**			**Right hem: ASD** **>** **TD**		
0.00022	81	100% pre-central	<0.00001	208	65% parahippocampal
0.00026	69	100% pars opercularis			35% entorhinal
0.00019	58	60% superior temporal	0.00043	97	100% superior temporal
		38% insula	0.00008	88	53% superior temporal
0.00038	45	100% pre-central			47% insula
0.00027	29	100% bankssts	0.00005	56	57% insula
0.00062	28	43% pre-central			43% transverse temporal
		36% caudal middle frontal	0.00016	52	100% superior temporal
		21% superior frontal	<0.00001	33	100% post-central
**Left hem: ASD** **<** **TD**			**Right hem: ASD** **<** **TD**		
0.00035	49	100% supramarginal	0.00092	23	100% pre-cuneus
0.00092	45	100% rostral middle frontal			
0.00007	29	100% lingual			
**CA vs. TD** ** Left hem: ASD** **>** **TD**			**Right hem: ASD** **>** **TD**		
0.00045	35	69% inferior parietal	0.00048	71	83% inferior parietal
		31% supramarginal			17% superior parietal
			0.00033	34	94% middle temporal
					6% inferior temporal
**Left hem: ASD** **<** **TD**			**Right hem: ASD** **<** **TD**		
0.00057	33	67% lateral occipital	0.00028	23	83% middle temporal
		18% inferior parietal			17% bankssts
		15% superior parietal			
0.00024	29	100% inferior temporal			
**RRSB vs. TD** ** Left hem: ASD** **>** **TD**			**Right hem: ASD** **>** **TD**		
0.00002	128	100% bankssts	<0.00001	165	68% insula
0.00091	42	100% pars triangularis			32% superior temporal
	26	92% paracentral	0.00008	103	77% post-central
		8% superior frontal			23% pre-central
			0.00025	44	100% pars triangularis
**Left hem: ASD** **<** **TD**			**Right hem: ASD** **<** **TD**		
0.00124	30	70% superior parietal	0.00013	38	82% precuneus
		30% pre-cuneus			18% superiorparietal
			0.00008	38	82% lingual
					18% lateraloccipital

*bankssts, the banks superior temporal sulcus. The results were corrected by vertex level p < 0.001 and cluster level FWE p < 0.05. The corrected and survived clusters were reported as % overlap with the Desikan–Killiany DK40 atlas*.

### Developmental Trajectories of Gyrification in ASD and TD

#### Cross-Sectional Regression

**Figure 3:**
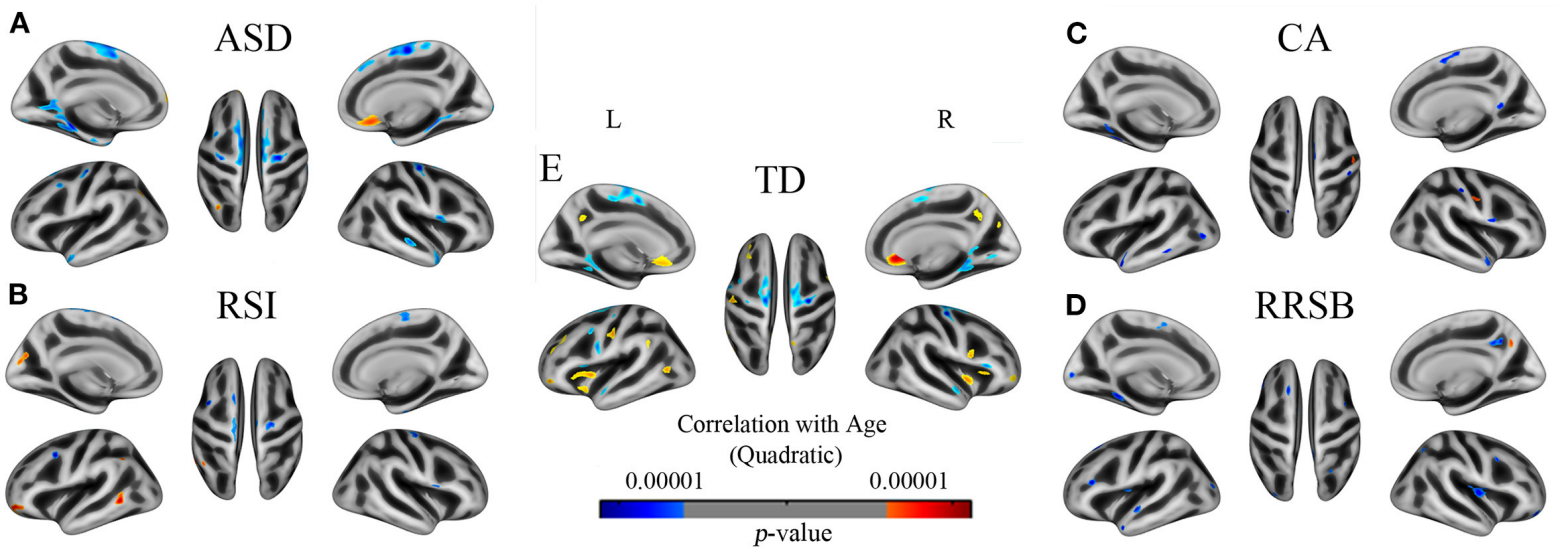
The developmental trajectory of the GI in subjects of ASD **(A)**, TD **(B)**, RSI **(C)**, CA **(D)**, and RRSB **(E)**. Cool and warm colors indicate the negative and positive correlations between age and gyrification, respectively. The results were corrected by vertex level *p* < 0.001 and cluster level FWE *p* < 0.05.

Beyond the above inquiries on intergroup differences, we further conducted regression analyses between GI and age to determine the developmental trajectories of gyrification in ASD and TD. Both the general ASD and TD groups exhibited mixed patterns of positive and negative associations between age and GI; however, they were disparately distributed across the cortical regions (Figure 3 and Supplementary Table 2).

Across the general ASD and TD groups, the negative correlations between GI and age almost showed in overlapped clusters, which were located in the left superior frontal, parahippocampal, isthmus cingulate, superior temporal, and pre-central cortices (*p* < 0.001) and the right superior frontal, pre-central, superior temporal, parahippocampal, and pars opercularis cortices (*p* < 0.001). However, our results revealed significant regional differences between the ASD and TD groups in the positive correlations of GI and age. The TD group showed positive correlations between age and GI in the left insula, post-central, pre-central, rostral anterior cingulate, rostral middle frontal, and pre-cuneus cortices (*p* < 0.001) and the right rostral anterior cingulate, medial frontal, insula, pre-central, and pars triangular cortices (*p* < 0.001). However, there was only one big cluster that exhibited positive correlations of GI and age in the ASD group, which was located in the right medial orbitofrontal and rostral anterior cingulate cortices (*p* < 0.001).

Furthermore, we found specific gyrification developmental trajectories in the three subgroups, which differ from the general ASD and TD groups. Especially the positive effects of age in gyrification, the RSI group exhibited in the left rostral middle frontal, middle temporal, cuneus, and inferior parietal cortices, the CA group exhibited in the right post-central cortex, and the RRSB group exhibited in the right pre-cuneus cortex.

#### Longitudinal Comparisons

**Figure 4:**
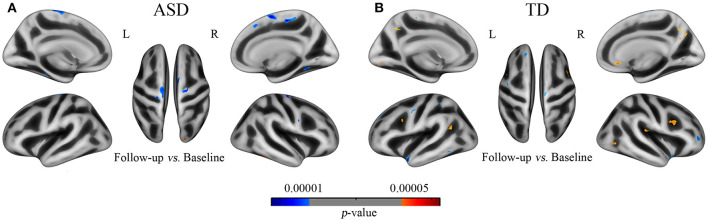
The developmental trajectory of the GI in subjects of ASD **(A)** and TD **(B)** from the longitudinal data (UCLA). Cool and warm colors indicate the negative and positive correlations between age and gyrification, respectively. The results were corrected by vertex level *p* < 0.001 and cluster level FWE *p* < 0.05.

We found that the results in longitudinal data also revealed the abnormities of gyrification developmental trajectory in ASD and the direction and regions of this group difference are basically consistent with the multiple regression analyses in cross-sectional data (Figure 4 and Supplementary Table 3). By comparing the GI between baseline and follow-up, we discovered that signification differences exhibited in 10 clusters in ASD, with 9 clusters of decreased gyrification located in the bilateral superior frontal, para- and pre-central, and fusiform cortices and 1 cluster of increased gyrification in the lateral occipital cortices (*p* < 0.001). The TD group showed significantly decreased gyrification in 8 clusters, which were located in the left superior frontal, superior temporal, superior parietal, and precentral and right insula and rostral middle frontal cortices (*p* < 0.001), and 11 clusters with increased gyrification in the left inferior parietal and pre-cuneus and right caudal middle frontal cortices (*p* < 0.001).

### Behavior–Brain Correlations

Several significant associations were found between the GI within significant clusters and clinical variables in the subgroups of ASD. Specifically, the GI in the left lingual gyrus was negatively correlated with scores in the ADI-R Social domain (*p* = 0.047, *d* = −0.23), but the GI in the right superior temporal cortex was positively correlated with scores in the ADI-R Social domain (*p* = 0.035, *d* = 0.25); lower scores in the ADI-R Communication domain were correlated with higher GI in the left lateral occipital cortex (*p* = 0.023, *d* = −0.26); and scores in the ADI-R RRSB domain score were correlated with higher GI in the right cuneus cortex (*p* = 0.030, *d* = −0.25) (Supplementary Table 5).

### Symptom-Defined Co-Activation Meta-Analytical Maps

**Figure 5:**
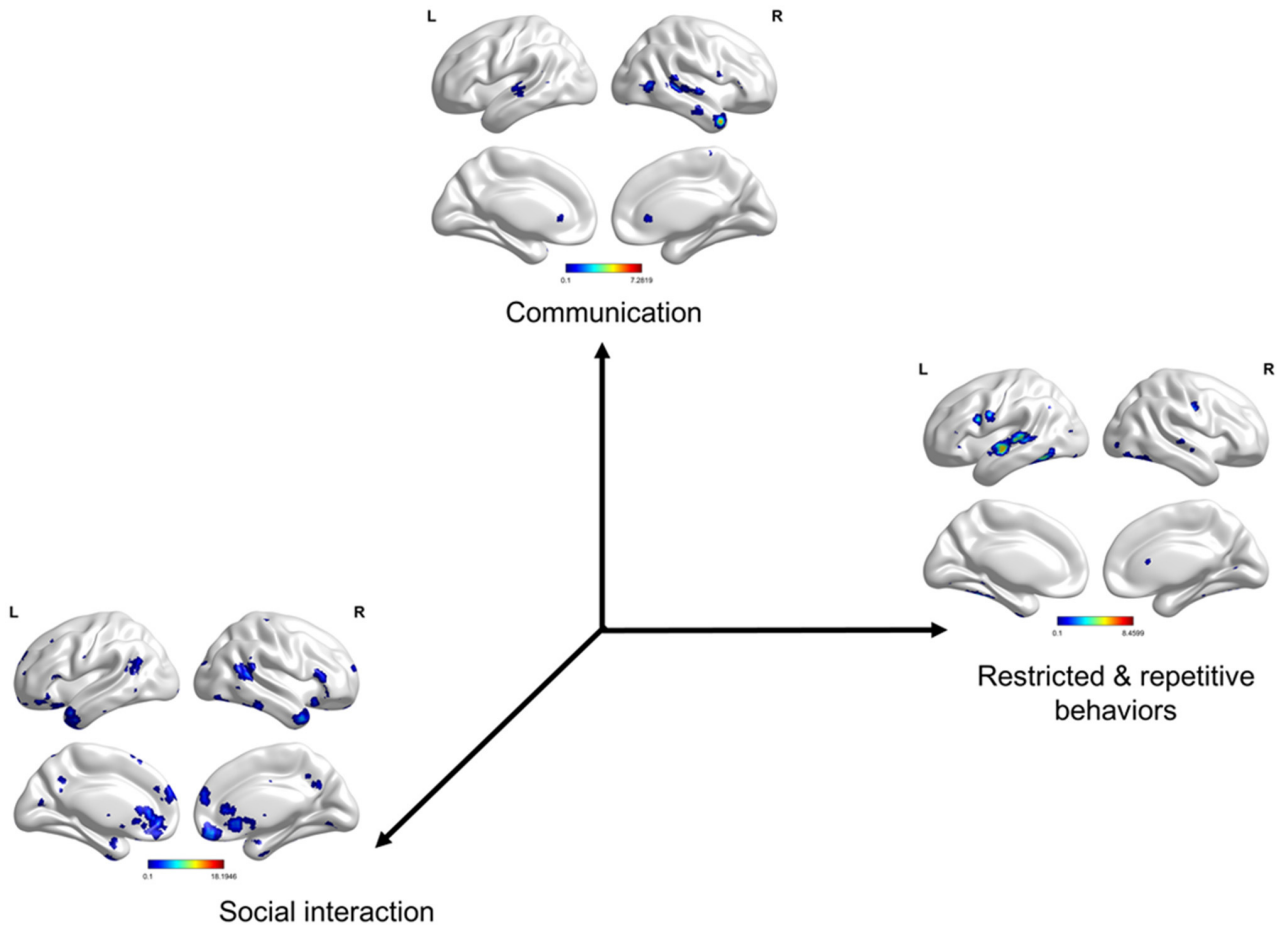
Symptom-defined co-activation meta-analytical maps. Social interaction-activated brain regions are located in the left anterior cingulate, orbital frontal, superior temporal, inferior temporal cortices, and rectus gyrus and the right superior frontal, inferior frontal, insula, superior occipital, pre-cuneus, temporal pole, superior temporal, inferior temporal cortices, and rectus gyrus. Communication-activated brain regions are located in the right temporal pole, superior temporal, and middle temporal cortices. Restricted and repetitive behavior-activated brain regions are located in the left superior temporal, middle temporal, pre-central, and fusiform cortices. The results were corrected by voxel-wise FDR *p* < 0.001 and cluster size >50 voxels.

The three meta-analytical maps clearly depict brain regions (or circuits) that evolved in each term (core symptom) (Figure 5 and Supplementary Table 4). We found the atypical patterns of gyrification in the three subgroups to be almost consistent with the three meta-analytical maps. Social interaction-activated brain regions are located in the left anterior cingulate, frontal, and temporal cortices and the right frontal, insula, occipital, pre-cuneus, and temporal cortices. Communication-activated brain regions are located in the right temporal cortex. Restricted and repetitive behavior-activated brain regions are located in the left temporal and frontal cortices.

### ROI-Wise Result Validation

To ensure that our results of vertex-wise comparisons are robust, we conducted ROI-wise analysis within the Desikan–Killiany 40 atlas parcellation with LMM with site as a random factor in SPSS 20.0. The ROI results are generally consistent with the vertex-level cortical findings, suggesting that our results are robust (Supplementary Table 5).

## Discussion

In this study, we used a large neuroimaging database (ABIDE I) to firstly investigate the general differences in cortical gyrification between individuals with ASD and matched TD controls. Then, we divided these ASD individuals into three subgroups (i.e., RSI, RRSB, and CA subgroups) based on their core symptoms to explore the subgroup differences of cortical gyrification. The subgroup differences showed both convergent and divergent cortical foci as the general comparison (i.e., ASD vs. TD). Furthermore, ASD in each subgroup exhibited regionally different developmental trajectories of the GI. Subsequently, we conducted a meta-analytical co-activation to constrain the core-symptom-related topographies. These findings suggest the variety of clinical presentations clearly relevant to the neuroanatomical heterogeneity of ASD, which can definitely help further clinical subgrouping, differential diagnosis, and heterogeneity classification of ASD.

### The Atypical Pattern of Gyrification in ASD

Autism spectrum disorder is a highly heterogeneous disease, especially in terms of brain structure (29). Numerous studies have reported increased gyrification in several cortical foci, including paracentral (6, 10), cingulate (10, 11), occipital (5, 6), and temporal cortices (11) in children and adolescents with ASD. In contrast, other studies found decreased gyrification in the parietal (6) and supramarginal gyrus (13) in adults with ASD and temporo-parietal junction in children with ASD (14, 15). Our results revealed likewise atypical patterns of gyrification in ASD, in directions overlapping with previous studies, but of varying regions and magnitude. For example, significantly higher GI in the bilateral temporal, right parahippocampal gyrus, and left frontal regions, which is generally consistent with previous studies that have reported ASD, exhibited higher gyrification in the frontal and temporal lobes (6, 12). Furthermore, significantly lower GI in two clusters of the bilateral occipital lobes in ASD is generally comparable with prior reports (12, 15). Despite failing to fully replicate the cortical foci that have been reported in previous studies, our findings support the view that neuroanatomical heterogeneity might be one basic trait of ASD (25). Given the high heterogeneity of the imaging studies, it is important to figure out the source of these differences in order to find reliable and objective biological markers for the diagnosis and prognosis of ASD.

### Regional GI Differences Showed in Subgroups of ASD

The degree of cortical folding is related to clinical phenotypes of ASD (30), yet studies exploring the relationships between clinical symptoms and neuroanatomical heterogeneity are rare. Previous investigations suggest that age (6), site (31), and gender (32) are primary sources of this inconsistency. However, a recent study found that ASD patients with different clinical symptom severity levels still showed inconsistent neuroanatomical difference patterns after controlled gender, age, site, and FIQ (25). Furthermore, Duret et al. revealed abnormal GI in the left fusiform and right temporal regions anchored with language performance, depending on speech onset, in patients with ASD (12). Consistent with those reports, we found distinct patterns of gyrification across the core-symptom-based subgroups (RSI, CA, and RRSB subgroups). Our results suggested that the different clinical symptoms across ASD subjects may contribute to the observed neuroanatomical heterogeneity.

Dividing the heterogeneous ASD cohort into symptom subgroups would increase the specificity of results in neuroimaging research findings (12, 25, 29). Compared with controls, the RSI subgroup exhibited significantly increased GI in the right superior temporal cortex that was one of the key components of the “social brain” (2). The length of the right superior temporal sulcus is related to emotional and social processing (33), and Kana et al. found that during an implicit emotion processing functional MRI (fMRI) task, ASD demonstrated significantly less activation in the superior temporal cortex and paralleling empathy quotient (34, 35). In the CA subgroup, we revealed increased GI in the right middle temporal and bilateral inferior parietal cortices, belonging to the speech processing network (36). Consistent with our results, a previous study found abnormal GI in the right temporal regions anchored with language performance, depending on speech onset, in patients with ASD (12). The RRSB subgroup showed increased gyrification in the left superior temporal sulcus and decreased gyrification in the left superior parietal cortex. These results are consistent with previous studies that have reported cortical thickness and volumes in the temporal lobe (37, 38) negatively correlate with the severity of repetitive behaviors/stereotyped patterns in patients with ASD. Topographically, the superior temporal sulcus and superior parietal cortex are located across the default mode and dorsal attention networks (39, 40). Abnormal integrations between the two large-scale networks and other systems, including visual and fine motor, have been reported and associated with restricted and repetitive behavior in ASD (41). Our results suggest that specific differences across subgroups are greatly overshadowed by considerable within-group variability apparent in the ASD group.

### Atypical Developmental Trajectories of Gyrification in ASD and Subgroups of ASD

The atypical gyrification patterns in ASD persist from childhood to adulthood (6, 7, 10). During ontogeny, cortical gyrification emerges first in the superior temporal, occipital, and parietal cortices (3). Our results revealed regionally different GI developmental trajectories across a wide age span in the ASD and TD groups, especially in those regions, which indicates that the abnormal patterns of gyrification in ASDs may start from prenatal to adulthood. Generally, the degree of brain folding increased in childhood and decreased in adolescence and adulthood across individuals with ASD and healthy controls and with different timing and speeds of changing among brain regions (3, 6, 42). In our study, most of the clusters showed significant negative effects of age in the GI that were located in the occipital, parietal, and temporal regions across the ASD and TD groups, which were consistent with previous studies (12). In the general population, the gyrification is greatest in the prefrontal and parieto-occipito-temporal association cortices (43), which is reflected in this study, that is, the positive correlation of age and GI existed in these regions in TD. However, ASD showed positive correlations with age only in the prefrontal cortex, which indirectly supports cerebral overgrowth at an early age but abnormally decreases and possibly degenerates across later childhood and adolescence in ASD (6).

The atypical developmental trajectories of brain structures encode positive psychotic symptoms (44). We examined gyrification developmental trajectory in the three subgroups of ASD and found that the brain folding difference patterns across the subgroups were also exhibited in the developmental trajectory, even more than the regional differences. The diverse findings of brain development have become a major hamper to understanding the pathogenesis of ASD (10, 45). Our results suggest that the inter-subject variance in clinical presentations may contribute to the heterogeneity of gyrification developmental trajectory across subjects with ASD.

### Limitations

In the current study, due to inclusion criteria and cross-group matching, many individuals with ASD failed to be included in subgroups. To maximize the sample size, we performed three matches between controls and ASD within each site based on age and gender for three subgroups, which resulted in partial overlaps of subjects between the controls. We used several templates designed for different ages in pre-processing for cross-age comparisons from children to early adulthood. Although there are slight differences between representative templates of different ages, they have been reduced to a very low level. Furthermore, our data come from multiple research centers around the world, which resulted in center-dependent clinical behavioral evaluation variations of ASD. However, the bias of this evaluation has been minimized by controlling the weight of different research centers. Because our study involves across-analysis, it seems more reasonable to further consider multiple corrections on this basis, similar to the practice of the Bonferroni correction. However, since our original analysis has considered FWE correction, further application of multiple corrections across the analysis seems to have less effect on the final result. Finally, the symptom-defined co-activation meta-analytical maps were based on the data by keyword retrieval that involved both pathological and physiological cases. There are some possibilities that the major symptom dimensions of ASD describe more pathological situations, which may be different from physiological results, that we cannot further clarify due to the influence of the number of documents.

## Conclusion

Our results further support that atypical patterns and developmental trajectories of gyrification exist in the autistic brain and may be the neuroanatomical substrate of pathogenesis for ASD. Furthermore, core-symptom-based classification can find more subtle changes in gyrification, which promotes the understanding of the neuroanatomical basis and the development of neuroimaging classifiers for differential diagnosis, and prognosis, even further treatment stratification for ASD.

## Data Availability Statement

The datasets presented in this study can be found in online repositories. The names of the repository/repositories and accession number(s) can be found in the article/Supplementary Material.

## Author Contributions

All authors listed have made a substantial, direct and intellectual contribution to the work, and approved it for publication.

## Conflict of Interest

The authors declare that the research was conducted in the absence of any commercial or financial relationships that could be construed as a potential conflict of interest.
