# Supplementary material for: Core-Symptom-Defined Cortical Gyrification Differences in Autism Spectrum Disorder
Source: Front Psychiatry. 2021 Apr 20;12:619367. doi: 10.3389/fpsyt.2021.619367 (PMC8093770; doi:10.3389/fpsyt.2021.619367)
Supplement: Supplementary file 1 [file Data_Sheet_1.docx]

**Table SI.** Scan Parameters for sites in ABIDE.

| Sites | Manufacturer | Model | Field Strength | Sequence | Flip Angle | TR/TE/TI (ms) | Voxel Size (mm) | Slice Orientation |
| --- | --- | --- | --- | --- | --- | --- | --- | --- |
| KKI | Philips | Achieva | 3T | FFE | 8 | 8.0/3.7/843 | 1x1x1 | Coronal |
| NYU | Siemens | Allegra | 3T | MPRAGE | 7 | 2530/3.25/1100 | 1.3x1.0x1.3 | Sagittal |
| PITT | Siemens | Allegra | 3T | MPRAGE | 7 | 2100/3.93/1000 | 1.1x1.1x1.1 | Sagittal |
| Stanford | GE | Signa | 3T | SPGR | 15 | 8.4/1.8/… | 0.86x1.5x0.86 | … |
| Trinity | Philips | Achieva | 3T | FFE | 8 | 8.5/3.9/1060.17 | 1x1x1 | Sagittal |
| UCLA | Siemens | Trio Tim | 3T | MPRAGE | 9 | 2300/2.84/853 | 1x1x1.2 | Sagittal |
| UM | GE | Signa | 3T | SPGR | 15 | 250/1.8/500 | 1x1x1.2 | Sagittal |
| Yale | Siemens | Trio Tim | 3T | MPRAGE | 9 | 1230/1.73/624 | 1x1x1 | Sagittal |

KKI: Kennedy Krieger Institute, Baltimore; NYU: NYU Langone Medical Center, New York; PITT: University of Pittsburgh School of Medicine; Stanford: Stanford University; Trinity: Trinity Centre for Health Sciences; UCLA: University of California, Los Angeles; UM: University of Michigan; Yale: Yale Child Study Center; “…”: missing data.

**Table S2.** Developmental trajectories of gyrification.

| RSI | | | CA | | | RRSB | | |  |
| --- | --- | --- | --- | --- | --- | --- | --- | --- | --- |
| *p*-value | Size | Overlap of atlas region | *p*-value | Size | Overlap of atlas region | *p*-value | Size | Overlap of atlas region | |
| Left hem: Positive correlation | | | | | | | | |  |
| 0.00004 | 120 | 43% rostral middle frontal |  |  |  |  |  |  |  |
|  |  | 37% pars orbitalis |  |  |  |  |  |  |  |
|  |  | 20% lateral orbitofrontal |  |  |  |  |  |  |  |
| 0.00001 | 81 | 65% middle temporal |  |  |  |  |  |  |  |
|  |  | 35% bankssts |  |  |  |  |  |  |  |
| 0.0003 | 65 | 89% cuneus |  |  |  |  |  |  |  |
|  |  | 11% precuneus |  |  |  |  |  |  |  |
| 0.00026 | 29 | 79% inferior parietal |  |  |  |  |  |  |  |
|  |  | 21% supramarginal |  |  |  |  |  |  |  |
| Left hem: Negative correlation | | | | | | | | |  |
| 0.00165 | 94 | 47% precentral | 0.00031 | 53 | 100% superior temporal | 0.00009 | 77 | 64% para hippocampal |  |
|  |  | 32% superior frontal | 0.00212 | 42 | 90% lingual |  |  | 21% fusiform |  |
|  |  | 21% paracentral |  |  | 10% fusiform |  |  | 16% lingual |  |
| 0.00001 | 51 | 100% caudal middle frontal | 0.00023 | 35 | 100% lateral occipital | 0.00111 | 52 | 48% precentral |  |
| 0.00025 | 45 | 100% superior frontal | 0.00011 | 32 | 100% fusiform |  |  | 42% insula |  |
|  |  |  | 0.00033 | 21 | 90% middle temporal |  |  | 10% postcentral |  |
|  |  |  |  |  | 10% inferior temporal | 0.00028 | 52 | 100% superior temporal |  |
|  |  |  | 0.00013 | 19 | 100% superior parietal | 0.00021 | 43 | 100% superior frontal |  |
|  |  |  |  |  |  | 0.00003 | 42 | 90% rostral middle frontal |  |
|  |  |  |  |  |  |  |  | 10% pars opercularis |  |
|  |  |  |  |  |  | 0.00011 | 29 | 86% superior temporal |  |
|  |  |  |  |  |  |  |  | 14% middle temporal |  |
|  |  |  |  |  |  | 0.0007 | 28 | 75% lateral occipital |  |
|  |  |  |  |  |  |  |  | 25% inferior parietal |  |
|  |  |  |  |  |  | 0.0017 | 27 | 100% superior frontal |  |
|  |  |  |  |  |  | 0.0002 | 26 | 100% pericalcarine |  |
| Right hem: Positive correlation | | | | | | | | |  |
|  |  |  | 0.00106 | 42 | 62% postcentral | 0.00052 | 27 | 100% precuneus |  |
|  |  |  |  |  | 38% precentral |  |  |  |  |
| Right hem: Negative correlation | | | | | | | | |  |
| 0.00007 | 96 | 100% precentral | 0.00064 | 77 | 91% superior frontal | 0.00005 | 181 | 69% insula |  |
| 0.00051 | 70 | 99% paracentral |  |  | 9% paracentral |  |  | 20% postcentral |  |
|  |  | 1% superior frontal | 0.00151 | 54 | 94% superior temporal |  |  | 8% precentral |  |
| 0.00039 | 26 | 54% pars opercularis |  |  | 6% middle temporal |  |  | 3% supramarginal |  |
|  |  | 46% precentral | 0.00012 | 45 | 100% postcentral | 0.00013 | 65 | 78% precuneus |  |
| 0.00075 | 26 | 88% fusiform | 0.00062 | 42 | 100% precentral |  |  | 22% isthmus cingulate |  |
|  |  | 12% entorhinal | 0.00046 | 33 | 91% isthmus cingulate | 0.00014 | 38 | 55% lateral orbitofrontal |  |
|  |  |  |  |  | 9% precuneus |  |  | 45% rostral middle frontal |  |
|  |  |  |  |  |  | 0.00105 | 23 | 100% superior parietal |  |
|  |  |  |  |  |  | 0.00011 | 20 | 100% caudal middle frontal |  |

**Table S****2** (Continued).

| ASD | | |  | TD | | |  |  |
| --- | --- | --- | --- | --- | --- | --- | --- | --- |
| *p*-value | Size | Overlap of atlas region |  | *p*-value | Size | Overlap of atlas region |  |  |
| Left hem: Positive correlation | | | | | | | | |
| 0.00005 | 60 | 100% inferior parietal |  | 0.00003 | 111 | 77% insula | | |
| 0.00184 | 28 | 100% superior frontal |  |  |  | 16% pars triangularis | | |
|  |  |  |  | 0.00008 | 99 | 62% postcentral | | |
|  |  |  |  |  |  | 38% precentral | | |
|  |  |  |  | 0.00021 | 80 | 73% rostral anterior cingulate | | |
|  |  |  |  |  |  | 25% medial orbitofrontal | | |
|  |  |  |  | 0.00054 | 71 | 96% insula | | |
|  |  |  |  |  |  | 4% lateral orbitofrontal | | |
|  |  |  |  | 0.0001 | 36 | 39% inferior parietal | | |
|  |  |  |  |  |  | 36% middle temporal | | |
|  |  |  |  |  |  | 25% lateral occipital | | |
|  |  |  |  | 0.00004 | 35 | 57% pars orbitalis | | |
|  |  |  |  |  |  | 43% rostral middle frontal | | |
|  |  |  |  | 0.0018 | 34 | 100% rostral middle frontal | | |
|  |  |  |  | 0.00047 | 30 | 100% precuneus | | |
|  |  |  |  | 0.00122 | 27 | 100% supramarginal | | |
|  |  |  |  | 0.00099 | 26 | 100% rostral middle frontal | | |
| Left hem: Negative correlation | | | | | | | |  |
| 0.00003 | 499 | 84% superior frontal |  | < 0.00001 | 452 | 70% superior frontal | | |
|  |  | 9% paracentral |  |  |  | 18% paracentral | | |
|  |  | 8% precentral |  |  |  | 12% precentral | | |
| < 0.00001 | 245 | 67% para hippocampal |  | 0.00057 | 116 | 74% para hippocampal | | |
|  |  | 20% lingual |  |  |  | 12% isthmus cingulate | | |
|  |  | 9% fusiform |  |  |  | 12% lingual | | |
|  |  | 2% isthmus cingulate |  | 0.00005 | 62 | 100% precentral | | |
| 0.00017 | 161 | 60% isthmus cingulate |  | 0.00024 | 42 | 100% pars opercularis | | |
|  |  | 40% lingual |  | 0.00017 | 35 | 100% caudal middle frontal | | |
| 0.00018 | 114 | 45% superior temporal |  | 0.00067 | 34 | 53% lingual | | |
|  |  | 38% temporal pole |  |  |  | 24% fusiform | | |
|  |  | 12% middle temporal |  |  |  | 24% para hippocampal | | |
|  |  | 4% entorhinal |  | 0.00059 | 30 | 100% superior temporal | | |
|  |  | 2% fusiform |  | 0.00042 | 23 | 100% precentral | | |
| 0.00001 | 84 | 100% precentral |  |  |  |  | | |
| 0.00012 | 43 | 100% caudal middle frontal |  |  |  |  | | |
| 0.00042 | 31 | 100% entorhinal |  |  |  |  | | |
| 0.00139 | 19 | 100% lingual |  |  |  |  | | |
| Right hem: Positive correlation | | | | | | | | |
| 0.00002 | 91 | 65% medial orbitofrontal |  | < 0.00001 | 79 | 80% rostral anterior cingulate | | |
|  |  | 35% rostral anterior cingulate |  |  |  | 20% medial orbitofrontal | | |

**Table S2** (Continued).

| ASD | | |  | TD | | |  |
| --- | --- | --- | --- | --- | --- | --- | --- |
| *p*-value | Size | Overlap of atlas region |  | *p*-value | Size | Overlap of atlas region |  |
| Right hem: Positive correlation | | | | | | | |
|  |  |  |  | 0.00002 | 76 | 100% insula | |
|  |  |  |  | 0.00009 | 56 | 71% precentral | |
|  |  |  |  |  |  | 29% pars opercularis | |
|  |  |  |  | 0.00086 | 50 | 100% precuneus | |
|  |  |  |  | 0.0009 | 41 | 98% rostral middle frontal | |
|  |  |  |  |  |  | 2% pars orbitalis | |
|  |  |  |  | 0.00039 | 31 | 81% pars triangularis | |
|  |  |  |  |  |  | 16% insula | |
|  |  |  |  |  |  | 3% pars opercularis | |
|  |  |  |  | 0.00047 | 29 | 76% superior parietal | |
|  |  |  |  |  |  | 24% precuneus | |
|  |  |  |  | 0.00032 | 20 | 100% precuneus | |
| Right hem: Negative correlation | | | | | | | |
| < 0.00001 | 378 | 63% superior frontal |  | < 0.00001 | 363 | 67% precentral | |
|  |  | 28% paracentral |  |  |  | 33% superior frontal | |
|  |  | 9% precentral |  | 0.00003 | 190 | 92% para hippocampal | |
| < 0.00001 | 165 | 100% precentral |  |  |  | 5% lingual | |
| 0.00021 | 134 | 49% superior temporal |  |  |  | 1% fusiform | |
|  |  | 30% temporal pole |  | 0.00081 | 61 | 98% isthmus cingulate | |
|  |  | 21% middle temporal |  |  |  | 2% para hippocampal | |
| 0.00003 | 125 | 75% para hippocampal |  | 0.00002 | 55 | 67% pars triangularis | |
|  |  | 22% lingual |  |  |  | 33% pars opercularis | |
| 0.00003 | 120 | 52% precentral |  | 0.00036 | 53 | 100% superior frontal | |
|  |  | 48% pars opercularis |  | 0.0004 | 53 | 100% superior temporal | |
| 0.00035 | 65 | 100% superior frontal |  | 0.00065 | 30 | 100% lingual | |
| 0.00038 | 60 | 100% paracentral |  |  |  |  | |
| 0.00006 | 58 | 69% middle temporal |  |  |  |  | |
|  |  | 31% superior temporal |  |  |  |  | |
| 0.00091 | 22 | 91% lateral occipital |  |  |  |  | |
|  |  | 9% pericalcarine |  |  |  |  | |

ASD: patients with autism spectrum disorder; TD: typically developing controls; RSI: impairments in reciprocal social interaction as the most serious in the three symptoms; CA: communication abnormalities as the most serious in the three symptoms; RRSB: stereotyped patterns of behavior as the most serious in the three symptoms; bankssts: banks superior temporal sulcus. The results were corrected by vertex level *p* <0.001 and cluster level FWE *p* <0.05. The corrected and survived clusters were reported as % overlap with the Desikan-Killiany DK40 atlas.

**Table S3.** Comparison of gyrification between baseline and follow-up in ASD and TD.

| *p*-value | Size | Overlap of atlas region | *p*-value | Size | Overlap of atlas region |
| --- | --- | --- | --- | --- | --- |
| UCLA  Left hem: ASD increasing | | | Left hem: TD increasing | | |
|  |  |  | 0.00027 | 32 | 100% inferior parietal |
|  |  |  | 0.0027 | 12 | 100% precuneus |
|  |  |  | 0.00004 | 10 | 80% lingual |
|  |  |  |  |  | 20% lateral occipital |
|  |  |  | 0.00074 | 9 | 100% lingual |
|  |  |  | 0.00166 | 7 | 100% caudal middle frontal |
|  |  |  | 0.00114 | 3 | 100% superior temporal |
| Right hem: ASD increasing | | | Right hem: TD increasing | | |
| 0.00163 | 35 | 69% inferior temporal | 0.00053 | 40 | 48% caudal middle frontal |
|  |  | 31% fusiform |  |  | 28% precentral |
| 0.00053 | 22 | 100% lateral occipital |  |  | 25% pars opercularis |
|  |  |  | 0.00111 | 2 | 100% supramarginal |
|  |  |  | 0.00029 | 5 | 100% precuneus |
|  |  |  | 0.00296 | 11 | 100% precuneus |
|  |  |  | 0.00018 | 11 | 100% rostral anterior cingulate |
|  |  |  | 0.00117 | 12 | 100% precuneus |
|  |  |  | 0.00021 | 12 | 100% lateral occipital |
|  |  |  | 0.00037 | 34 | 100% supramarginal |
| Left hem: ASD decreasing | | | Left hem: TD decreasing | | |
| 0.00065 | 111 | 41% superior frontal | 0.0005 | 2 | 100% paracentral |
|  |  | 36% paracentral | 0.00094 | 8 | 100% inferior temporal |
|  |  | 23% precentral | 0.00023 | 10 | 100% superior parietal |
| 0.00028 | 19 | 100% precentral | 0.00183 | 15 | 100% precentral |
| 0.00175 | 14 | 100% fusiform | 0.00211 | 23 | 100% superior frontal |
|  |  |  | < 0.0001 | 67 | 100% superior temporal |
| Right hem: ASD decreasing | | | Right hem: TD decreasing | | |
| 0.00004 | 70 | 100% superior frontal | 0.00097 | 36 | 61% insula |
| 0.00007 | 54 | 100% precentral |  |  | 19% temporal pole |
| 0.00003 | 53 | 100% paracentral |  |  | 14% superior temporal |
| 0.00031 | 27 | 56% fusiform | 0.00014 | 30 | 100% rostral middle frontal |
|  |  | 41% lingual | 0.00119 | 27 | 89% insula |
|  |  | 4% para hippocampal | 0.00097 | 11 | 55% precentral |
| 0.0011 | 20 | 100% superior frontal |  |  | 45% paracentral |
| 0.0008 | 6 | 100% precentral | 0.00325 | 3 | 100% superior frontal |
| 0.00171 | 5 | 100% temporal pole |  |  |  |

UCAL: University California. The results were corrected by vertex level *p* <0.001 and cluster level FWE *p* <0.05. The corrected and survived clusters were reported as % overlap with the Desikan-Killiany DK40 atlas.

**Table S4.** Symptom-defined co-activation meta-analytic maps.

| Symptom | Region Label | Extent | *t*-value | MNI Coordinates | | |
| --- | --- | --- | --- | --- | --- | --- |
|  |  |  |  | x | y | z |
| Language-positive | Temporal_Pole_Mid_R | 52 | 7.282 | 48 | 10 | -34 |
|  | Temporal_Mid_R | 66 | 6.134 | 46 | -36 | 4 |
|  | Temporal_Sup_R | 29 | 6.044 | 70 | -36 | 10 |
|  | Temporal_Sup_R | 35 | 5.714 | 62 | -12 | 0 |
|  | Temporal_Mid_R | 23 | 5.025 | 48 | -62 | 6 |
| Repetition-positive | Temporal_Sup_L | 282 | 8.460 | -54 | -12 | -6 |
|  | Temporal_Mid_L | 282 | 6.280 | -60 | -30 | 6 |
|  | Location not in atlas | 133 | 6.062 | -38 | -54 | -8 |
|  | Cerebellum_Crus1_L | 133 | 6.001 | -50 | -58 | -26 |
|  | Cerebellum_Crus1_R | 93 | 6.009 | 44 | -72 | -20 |
|  | Precentral_L | 48 | 5.899 | -46 | 6 | 22 |
|  | Fusiform_L | 107 | 5.878 | -38 | -36 | -24 |
|  | Precentral_L | 24 | 5.739 | -58 | -2 | 28 |
| Social  interaction-positive | Location not in atlas | 161 | 10.763 | 6 | -8 | -40 |
|  | Location not in atlas | 161 | 5.689 | 12 | 8 | -26 |
|  | Location not in atlas | 305 | 9.083 | -32 | -12 | -46 |
|  | Temporal_Inf_L | 305 | 7.618 | -46 | 4 | -34 |
|  | Location not in atlas | 32 | 7.893 | 30 | -42 | 18 |
|  | Location not in atlas | 60 | 7.630 | -18 | 20 | 20 |
|  | Temporal_Mid_R | 247 | 7.507 | 64 | -56 | 12 |
|  | Temporal_Mid_R | 247 | 5.877 | 44 | -48 | 18 |
|  | Temporal_Pole_Mid_R | 156 | 7.415 | 50 | 14 | -40 |
|  | Rectus_R | 119 | 6.967 | 8 | 48 | -18 |
|  | Location not in atlas | 48 | 6.847 | 26 | 18 | 18 |
|  | Cerebellum_Crus1_R | 68 | 6.689 | 22 | -78 | -28 |

**Table S4** (Continued).

| Symptom | Region Label | Extent | *t*-value | MNI Coordinates | | |
| --- | --- | --- | --- | --- | --- | --- |
|  |  |  |  | x | y | z |
| Social interaction-positive | ACC_pre_L | 189 | 6.657 | -6 | 40 | 0 |
|  | Cerebellum_Crus2_L | 73 | 6.625 | -22 | -78 | -40 |
|  | Location not in atlas | 77 | 6.295 | -62 | -60 | 22 |
|  | ACC_sub_L | 57 | 6.232 | 2 | 20 | -8 |
|  | Frontal_Sup_Medial_R | 154 | 5.699 | 6 | 56 | 22 |
|  | Temporal_Inf_R | 37 | 5.689 | 58 | -36 | -28 |
|  | Precuneus_R | 38 | 5.670 | 4 | -60 | 32 |
|  | Temporal_Mid_L | 25 | 5.648 | -66 | -18 | -6 |
|  | Occipital_Sup_R | 36 | 5.624 | 28 | -76 | 16 |
|  | Rectus_L | 32 | 5.471 | -6 | 44 | -16 |
|  | OFCpost_L | 32 | 5.260 | -24 | 22 | -18 |
|  | Insula_R | 25 | 5.235 | 32 | 22 | -20 |
|  | Frontal_Inf_Tri_R | 33 | 5.204 | 50 | 28 | 10 |
|  | Location not in atlas | 25 | 5.167 | 34 | -62 | 12 |
|  | OFCant_L | 29 | 5.021 | -26 | 42 | -14 |

Note: Table shows all local maxima separated by more than 20 mm, *t* > 0.0001; *p* < Inf; df = Inf; minimum extent = 20. Regions were automatically labeled using the AAL3 atlas. x, y, and z =Montreal Neurological Institute (MNI) coordinates in the left-right, anterior-posterior, and inferior-superior dimensions, respectively.

**Table S5.** Region of interest (ROI)-wise result validation with linear mixed models.

| Regions | Variables | Estimate | F | *p-*value | *d* | |
| --- | --- | --- | --- | --- | --- | --- |
| ASD-TD Left hem | | | | | | |
| bankssts | group | 0.433953 | 6.97774 (1,665.524290) | 0.008447 | | 0.21 |
| lateral occipital | group | -0.195366 | 7.485279 (1,661.794805) | 0.006387 | | -0.21 |
| lateral orbitofrontal | group | -0.161082 | 3.989757 (1,661.190367) | 0.046187 | | -0.16 |
| rostral anterior cingulate | group | -0.256281 | 4.137911(1,663.938889) | 0.04233 | | -0.16 |
| lingual | age | 0.019072 | 4.131309 (1,664.994004) | 0.042494 | | 0.16 |
| caudal anterior cingulate | age2 | 0.003855 | 4.397464 (1,665.954885) | 0.036369 | | 0.16 |
| insula | group*age | 0.066027 | 8.721327 (1,660.605723) | 0.003257 | | 0.23 |
| paracentral | group*age2 | -0.005738 | 5.005314 (1,662.927827) | 0.025601 | | -0.17 |
| superior temporal | group*age | 0.043451 | 4.668832 (1,661.462019) | 0.031073 | | 0.17 |
| transverse temporal | group*age | 0.123301 | 9.611657 (1,663.666851) | 0.002015 | | 0.24 |
| ASD-TD Right hem | | | | | | |
| cuneus | group | -0.312943 | 7.476727 (1,661.502184) | 0.006417 | | -0.21 |
| entorhinal | group | 0.507163 | 8.729586 (1,660.414195) | 0.003242 | | 0.23 |
| lateral orbitofrontal | group | -0.27307 | 13.413359 (1,660.536716) | 0.00027 | | -0.29 |
| transverse temporal | group | 0.552727 | 5.148474 (1,664.321191) | 0.023587 | | 0.18 |
| entorhinal | age | 0.051098 | 8.912272 (1,661.550053) | 0.002937 | | 0.17 |
| fusiform | age | 0.019087 | 5.024683 (1,662.581730) | 0.025319 | | 0.17 |
| superior temporal | age | 0.019562 | 4.009049 (1,664.537056) | 0.045663 | | 0.16 |
| fusiform | age2 | 0.002157 | 4.764777 (1,662.834813) | 0.029398 | | 0.17 |
| transverse temporal | group*age | 0.105374 | 4.695345 (1,664.597203) | 0.030599 | | 0.17 |
| postcentral | group*age2 | -0.004377 | 4.526757 (1,663.378001) | 0.033737 | | -0.17 |
| rostral middle frontal | group*age2 | 0.003258 | 5.281249 (1,661.416908) | 0.021867 | | 0.18 |
| RSI-TD Left hem | | | | | | |
| bankssts | group | 0.509934 | 5.74746 (1,425.945792) | 0.016942 | | 0.23 |
| cuneus | age | 0.045811 | 4.936047 (1,242.496199) | 0.027226 | | 0.29 |
| entorhinal | age | -0.070728 | 4.876634 (1,413.552415) | 0.027771 | | -0.22 |
| frontal pole | group | 0.459822 | 4.316198 (1,423.528694) | 0.038353 | | 0.2 |
| frontal pole | age2 | -0.007435 | 4.346514 (1,372.144660) | 0.037766 | | -0.22 |
| inferior temporal | age2 | 0.003356 | 3.876091 (1,383.135278) | 0.049699 | | 0.2 |
| insula | age | 0.090205 | 24.901579 (1,406.746372) | 8.96E-07 | | 0.5 |
| isthmus cingulate | age | -0.059725 | 4.822637 (1,301.977460) | 0.028849 | | -0.25 |
| lingual | age | -0.032071 | 4.076449 (1,344.564430) | 0.044259 | | -0.22 |
| paracentral | age | -0.041585 | 5.259067 (1,210.679726) | 0.022819 | | -0.32 |
| para hippocampal | group | 0.738565 | 2.477368 (1,247.713584) | 0.016902 | | 0.23 |
| rostral middle frontal | age | 0.035623 | 10.4426 (1,329.757037) | 0.001356 | | 0.36 |
| superior parietal | age | 0.029596 | 5.009279 (1,296.064893) | 0.025955 | | 0.26 |
| isthmus cingulate | group*age | 0.106571 | 4.420936 (1,422.365785) | 0.036092 | | -0.31 |
| medial orbitofrontal | group*age2 | -0.008782 | 4.690248 (1,422.948440) | 0.030892 | | -0.21 |

**Table S5** (Continued).

| Regions | Variable | Estimate | F | *p-*value | *d* | |
| --- | --- | --- | --- | --- | --- | --- |
| RSI-TD Right hem | | | | | | |
| lateral orbitofrontal | group | -0.301761 | 10.206752 (1,422.632029) | 0.001504 | | 0.25 |
| middle temporal | group | 0.257351 | 5.738552 (1,422.904082) | 0.01703 | | 0.23 |
| para hippocampal | group | 0.637032 | 5.273936 (1,423.337538) | 0.022135 | | 0.22 |
| superior temporal | group | 0.276656 | 4.329562 (1,423.526760) | 0.038056 | | 0.2 |
| transverse temporal | group | 0.834684 | 7.306366 (1,423.679934) | 0.007147 | | 0.26 |
| entorhinal | age | -0.080829 | 6.311506 (1,423.655750) | 0.012366 | | -0.24 |
| fusiform | age | -0.033424 | 4.591433 (1,397.071862) | 0.032739 | | -0.22 |
| insula | age | 0.045959 | 6.306202 (1,414.201501) | 0.012411 | | 0.25 |
| isthmus cingulate | age | -0.066619 | 7.209798 (1,324.331252) | 0.007623 | | -0.3 |
| lateral orbitofrontal | age | 0.034406 | 6.767031 (1,418.829236) | 0.009615 | | 0.25 |
| lingual | age | -0.031249 | 3.969259 (1,367.223277) | 0.047078 | | -0.21 |
| paracentral | age | -0.049758 | 10.11911 (1,429.000000) | 0.001574 | | -0.31 |
| pars triangularis | age | 0.052331 | 10.450732 (1,247.785707) | 0.001392 | | 0.41 |
| precentral | age | -0.028349 | 5.44065 (1,324.458175) | 0.020285 | | -0.26 |
| precuneus | age | 0.023307 | 4.192954 (1,262.505425) | 0.041587 | | 0.25 |
| temporal pole | age | -0.057832 | 4.323019 (1,376.383395) | 0.038277 | | -0.21 |
| posterior cingulate | age2 | -0.004064 | 4.074715 (1,358.150751) | 0.044274 | | -0.21 |
| cuneus | group*age | 0.094564 | 6.405564 (1,420.305758) | 0.01174 | | 0.25 |
| lateral occipital | group*age | 0.048407 | 4.983947 (1,420.803732) | 0.026109 | | 0.22 |
| para hippocampal | group*age2 | -0.01966 | 5.16428 (1,420.784507) | 0.02356 | | -0.22 |
| rostral anterior cingulate | group*age2 | -0.011861 | 5.628609(1,419.515932) | 0.01812 | | -0.23 |
| CA-TD Left hem | | | | | | |
| lateral occipital | group | -0.388348 | 6.955924 (1,190.000000) | 0.009046 | | -0.38 |
| lingual | group | -0.430867 | 5.154754 (1,188.545748) | 0.024314 | | -0.33 |
| caudal anterior cingulate | age | -0.068581 | 3.913723 (1,189.687239) | 0.049341 | | -0.29 |
| insula | age | 0.099701 | 14.193633 (1,188.769379) | 0.00022 | | 0.55 |
| medial orbitofrontal | age | 0.054745 | 4.833993 (1,189.922023) | 0.029112 | | 0.32 |
| rostral anterior cingulate | age | 0.060312 | 4.453238 (1,189.573997) | 0.036147 | | -0.5 |
| precuneus | age2 | -0.009194 | 5.272292 (1,168.338222) | 0.0229 | | -0.35 |
| bankssts | group*age | -0.207329 | 5.504996 (1,188.000000) | 0.020004 | | -0.34 |
| inferior parietal | group*age2 | 0.02488 | 10.087934 (1,185.988143) | 0.001747 | | 0.47 |
| CA-TD Right hem | | | | | | |
| posterior cingulate | group | 0.514933 | 5.363215 (1,189.175127) | 0.021638 | | 0.34 |
| fusiform | age | -0.055434 | 6.380847 (1,189.312962) | 0.012355 | | -0.37 |
| insula | age | 0.072678 | 9.216296 (1, 188.486890) | 0.002738 | | 0.44 |
| isthmus cingulate | age | -0.076452 | 5.031941 (1,189.724205) | 0.026041 | | -0.33 |
| pars orbitalis | age | -0.074901 | 9.062763 (1,189.968906) | 0.002963 | | -0.44 |
| pericalcarine | age | -0.053877 | 4.127471 (1,188.540073) | 0.043596 | | -0.3 |
| precuneus | age | 0.051232 | 10.481411 (1,189.051962) | 0.001424 | | 0.47 |
| bankssts | age2 | 0.015575 | 4.550985 (1,190.000000) | 0.034183 | | 0.31 |
| pericalcarine | age2 | -0.012554 | 4.032078 (1,186.090971) | 0.046089 | | -0.29 |

**Table S5** (Continued).

| Regions | Variable | Estimate | F | *p-*value | *d* | |
| --- | --- | --- | --- | --- | --- | --- |
| CA-TD Right hem | | | | | | |
| rostral middle frontal | age2 | -0.00963 | 11.998844 (1,190.000000) | 0.000658 | | 0.31 |
| inferior temporal | group*age | -0.078122 | 4.275518 (1,186.249746) | 0.040047 | | -0.3 |
| middle temporal | group*age2 | -0.022373 | 6.795957 (1,185.959354) | 0.009878 | | -0.38 |
| RRSB-TD Left hem | | | | | | |
| bankssts | group | 1.231506 | 11.031858 (1,157.773359) | 0.001113 | | 0.53 |
| insula | group | 0.501799 | 4.161997 (1,156.431940) | 0.043023 | | -0.35 |
| lateral orbitofrontal | group | -0.382511 | 4.512939 (1,156.223269) | 0.035213 | | -0.34 |
| pars triangularis | group | 0.534334 | 4.553463 (1,158.000000) | 0.034396 | | 0.34 |
| para hippocampal | age | -0.128566 | 5.536887 (1,156.433649) | 0.019864 | | -0.38 |
| rostral anterior cingulate | age | 0.088328 | 7.197451 (1,157.827403) | 0.008079 | | 0.43 |
| superior temporal | age | -0.055157 | 4.910441 (1,156.452835) | 0.028138 | | -0.35 |
| cuneus | age2 | -0.008953 | 4.004964 (1,119.806364) | 0.047626 | | -0.37 |
| pericalcarine | age2 | -0.00964 | 5.217499 (1,157.986956) | 0.023692 | | -0.36 |
| posterior cingulate | group*age | -0.131868 | 4.587262 (1,154.040142) | 0.033783 | | -0.3 |
| para hippocampal | group*age2 | -0.030759 | 4.197786 (1,154.010541) | 0.042174 | | -0.33 |
| RRSB-TD Right hem | | | | | | |
| cuneus | group | -0.525182 | 3.928416 (1,158.000000) | 0.049211 | | -0.32 |
| lateral orbitofrontal | group | -0.473499 | 8.895556 (1,156.216614) | 0.003318 | | -0.48 |
| pars opercularis | group | 0.503778 | 4.60238 (1,156.646580) | 0.033469 | | 0.34 |
| fusiform | age | -0.051432 | 4.744709 (1,157.060739) | 0.03088 | | -0.35 |
| lingual | age | -0.046102 | 4.286255 (1,156.167579) | 0.040068 | | -0.33 |
| pars triangularis | age | 0.059194 | 5.270183 (1,157.481663) | 0.023013 | | 0.37 |
| rostral anterior cingulate | age | 0.06738 | 4.097255 (1,157.049855) | 0.044648 | | 0.32 |
| lingual | age2 | -0.007508 | 5.48451 (1,157.344618) | 0.020439 | | -0.37 |
| lingual | group*age2 | -0.014513 | 5.636285 (1,153.987973) | 0.018825 | | -0.38 |
| ASD Left hem | | | | | | |
| lateral occipital | communication | -0.038758 | 5.212832 (1, 297.679876) | 0.023125 | | -0.26 |
| lingual | social | -0.044955 | 3.994872 (1, 295.093703) | 0.046556 | | -0.23 |
| precuneus | social | -0.0368 | 5.928507 (1, 295.208628) | 0.01549 | | -0.28 |
| ASD Right hem | | | | | | |
| frontal pole | communication | -0.077509 | 4.531836 (1, 297.081708) | 0.034092 | | -0.35 |
| posterior cingulate | communication | 0.0564 | 5.164556 (1, 298.686538) | 0.023763 | | 0.26 |
| cuneus | RRB | -0.07828 | 4.742099 (1, 260.421561) | 0.030331 | | -0.25 |
| fusiform | RRB | 0.058629 | 4.622420 (1, 297.068515) | 0.032364 | | 0.25 |
| isthmus cingulate | RRB | -0.129472 | 8.816131 (1, 297.642023) | 0.003229 | | -0.34 |
| pars triangularis | RRB | 0.053998 | 6.284927 (1, 296.344457) | 0.012712 | | 0.29 |
| para hippocampal | social | -0.134827 | 6.787520 (1, 295.693747) | 0.009644 | | -0.3 |
| superior temporal | social | 0.136315 | 4.490133 (1, 298.974865) | 0.034915 | | 0.25 |

*d*: Cohen’s *d*; bankssts: banks superior temporal sulcus; age: linear effects of age; age2: quadratic effects of age; social: ADI-R-SOCIAL-TOTAL-A, Reciprocal Social Interaction Subscore (A) for Autism Diagnostic Interview-Revised; communication: ADI-R-VREBAL-TOTAL-BV, Abnormalities in Communication Subscore (B) for Autism Diagnostic Interview-Revised; RRB: ADI-R-RRB-TOTAL-C, Restricted, Repetitive, and Stereotyped Patterns of Behavior Subscore (A) for Autism Diagnostic Interview-Revised; Only significantly correlated regions were presented, *p* < 0.05 was chosen as the cut off value. Positive effects: estimate-value > 0; Negative effects: estimate< 0.
